# Supplementary material for: Sutureless versus Hand-Sewn Coronary Anastomoses: A Systematic Review and Meta-Analysis
Source: J Clin Med. 2022 Jan 29;11(3):749. doi: 10.3390/jcm11030749 (PMC8837108; doi:10.3390/jcm11030749)
Supplement: Supplementary file 1 [file jcm-11-00749-s001.zip › jcm-1557083-supplementary.pdf]

SUPPLEMENTARY DATA

Search strategy

A. Pubmed incl. MEDLINE

(coronary artery bypass graft*[Title/Abstract] OR CABG[Title/Abstract] OR coronary surgery[Title/Abstract] OR cardiovascular surger*[Title/Abstract] OR coronary vessel*[Title/Abstract] OR lad[Title/Abstract] OR Left anterior descending arter*[Title/Abstract] OR lima[Title/Abstract] OR "left internal mammarian"[Title/Abstract] OR "left internal mammary"[Title/Abstract] OR "left internal thoracic"[Title/Abstract] OR LITA[Title/Abstract] OR coronary arter*[Title/Abstract] OR "Coronary Artery Bypass"[Mesh] OR "Coronary Vessels"[Mesh])

AND

(device*[Title/Abstract] OR connector*[Title/Abstract] OR stapler*[Title/Abstract] OR instrument*[Title/Abstract] OR anastomotic technique*[Title/Abstract] OR experimental*[Title/Abstract] OR sutureless[Title/Abstract] OR facilitated[Title/Abstract] OR clip*[Title/Abstract] OR bonding[Title/Abstract] OR probe*[Title/Abstract] OR non-suture[Title/Abstract] OR nonsuture[Title/Abstract] OR stapl*[Title/Abstract] OR "Surgical Stapling"[Mesh] OR surgical staplers[MeSH])

AND

(hand-sutured[Title/Abstract] OR sutur*[Title/Abstract] OR conventional[Title/Abstract] OR anastom*[Title/Abstract] OR graft*[Title/Abstract] OR "Suture Techniques"[Mesh])

B. EMBASE

('coronary artery bypass graft*':ti,ab,kw OR 'CABG':ti,ab,kw OR 'coronary surgery':ti,ab,kw OR 'cardiovascular surger*':ti,ab,kw OR 'coronary vessel':ti,ab,kw OR 'lad':ti,ab,kw OR 'left anterior descending arter*':ti,ab,kw OR 'lima':ti,ab,kw OR 'left internal mammarian':ti,ab,kw OR 'left internal mammary':ti,ab,kw OR 'left internal thoracic':ti,ab,kw OR 'lita':ti,ab,kw OR 'coronary arter*':ti,ab,kw OR 'coronary vessels surgery'/exp OR 'coronary artery surgery'/exp)

AND

('device*':ti,ab,kw OR 'connector*':ti,ab,kw OR 'stapler*':ti,ab,kw OR 'instrument*':ti,ab,kw OR 'anasomotic technique*':ti,ab,kw OR 'experimental*':ti,ab,kw OR 'sutureless':ti,ab,kw OR 'facilitated':ti,ab,kw OR 'clip*':ti,ab,kw OR 'probe*':ti,ab,kw OR 'non-suture':ti,ab,kw OR 'nonsuture':ti,ab,kw OR 'stapl*':ti,ab,kw OR 'surgical stapling'/exp OR 'connector'/exp OR 'anastomotic device'/exp)

AND

('hand-sutured':ti,ab,kw OR 'sutur*':ti,ab,kw OR 'conventional':ti,ab,kw OR 'anastom*':ti,ab,kw OR 'graft*':ti,ab,kw OR 'suture technique'/exp OR 'vascular suture'/exp)

Table S1. PICOS-table on in- and exclusion criteria.

Parameter	Inclusion criteria	Exclusion criteria
Patients	Humane, adult (18 years or older) population	Animal or cadaveric trials, or >50% of the included population underwent emergent or reoperation CABG
Intervention	CABG with a sutureless device for the coronary anastomosis	
Control	CABG with a conventional hand-sewn coronary anastomosis	
Outcomes		
Study design	Controlled clinical trial<br>MREC approved the trial	

Abbreviations: CABG, coronary artery bypass grafting ; MREC, medical research ethics committee.

Table S2. Newcastle-Ottawa quality assessment for cohort studies.

	Selection				Comparability	Outcome			
**Study**	**Representative of the intervention group**	**Selection of the control group**	**Ascertainment of exposure**	**Outcome of interest not present at the start of the study**	**Comparability of cohorts on the basis of design, or analysis controlled for confounders**	**Assessment of outcomes**	**Sufficient follow-up time[a]**	**Adequacy of follow-up**	**Total (8/8)**
Eckstein 2002	*	*	*	*	*	*	*	*	8/8
Wiklund 2005	*	*	*	*	*	*	*	*	8/8
Klima 2003	*	*	*	*	*	*	0	*	7/8
Vicol 2006	*	*	*	*	*	*	*	0	7/8
Kim 2004	*	*	*	*	*	*	0	*	7/8
Klima 2005	*	*	*	*	*	*	*	0	7/8
Boening 2005	*	*	*	*	*	*	*	*	8/8
Cai 2007	*	0	*	*	0	*	*	0	5/8
Verberk-moes 2012	*	*	*	*	*	*	*	*	8/8
Balkhy 2018	*	0	*	*	*	*	*	*	7/8
Cheng 2021	*	*	*	*	*	*	*	*	8/8

a. Cut-off on a minimum 30-day follow-up.

Table S3. Study design.

Study	Device	Study type	Outcome retrieval	Randomization	Inclusion	Exclusion
Eckstein 2002	St Jude DAD	NRCT	Not blinded	Not randomized	First-time CABG with at least 1 anastomosis vein-coronary, coronary OD < 3mm and ID < 2.5mm, vein graft ID < 3.5mm.	-
Wiklund 2005	St Jude DAD	RCT	Not blinded	Randomized	Coronary OD > 2.5mm, coronary ID > 2.0mm, vein graft ID > 3.5mm.	Emergency procedures, reoperation CABG, recent neurologic events, significant comorbidities, LVEF < 25%, pregnancy or nursing, requirement of chronic anticoagulation other than acetylsalicylic acid .
Klima 2003	MVP	NRCT	Not blinded	Not randomized	Multivessel CAD, target coronary artery stenosis ≥ 80%, free of plaque or calcification at the target site, coronary ID > 2mm.	LVEF < 30%, contraindication to aspirin or clopidogrel, emergent CABG, redo CABG requiring additional noncoronary procedures.
Vicol 2006	MVP	NRCT	Not blinded	Not randomized	Target coronary artery with high-grade stenosis, coronary ID > 1.5mm, no atherosclerotic plaque at the target site.	Contraindication for antiplatelet therapy, in whom MRI is planned, patients who refuse implantation.
Kim 2004	AADD	NRCT	Not blinded	Not randomized	-	Failure of graft flaring onto the implant pins, small and diseased coronary target, conversion to CPB.
Klima 2005	CAC	NRCT	Not blinded	Not randomized	-	Severely sclerotic and calcified coronaries, tandem stenoses, too small or fragile-appearing vessels, vessels with inadequate flow distribution area, intramuscular segments, multiple-stented targets.
Boening 2005	CAC	NRCT	Not blinded	Not randomized	Between 35 and 80 years of age, informed consent, agrees to follow-up evaluation, no known contraindications for CAG, candidate for isolated CABG via median sternotomy and scheduled for at least one non-lad saphenous vein bypass graft, target coronary artery stenosis > 50% per CAG, vein graft ID 3.0 - 4.0mm and free of distal ligating clips or suture ligatures and sufficient length to cut off the connector in case of conversion, coronary ID > 2mm, proximal aortic anastomosis on connector-graft is hand-sewn and provides solely flow to the connector graft.	No informed consent, participating in other clinical investigation to device or drug, history of cerebrovascular disease, renal insufficiency (creat < 3.0mg/dl or dialysis daily), LVEF < 25%, history of malignant ventricular tachyarrhythmia, sustained VT or VF leading to cardiac arrest, contraindication to antiplatelet therapy that includes aspirin and clopidogrel, patient has had previous cardiac surgery, pregnant or nursing, HIV positive or hepatic failure, hemodynamic instability that requires inotropic support or a mechanical assist device, allergy to nickel, coronary target is severely calcified or contains soft atheroma anteriorly within 2mm proximally from the heel and 4mm distally from the toe, connector-graft is a Y-graft, connector graft is a sequential graft.

Table S3. Study design, continued.

Study	Device	Study type	Outcome retrieval	Randomization	Inclusion	Exclusion
Cai 2007	C-port	NRCT (historical control)	Not blinded	Not randomized	-	Poor vein grafts, severely diseased and small coronary targets < 1mm, any emergent procedures, need for IABP, any bleeding diathesis.
Verberk-moes 2012	C-port	RCT	Single blinded (participants)	Randomized	Informed consent, > 17 years of age, willing and able to have follow-up visits and examinations, EuroSCORE < 2, at least 2VD, target vessel free from severe calcifications, coronary target ID > 1.25mm and single wall thickness < 0.75mm, donor graft of suitable quality and single wall thickness < 0.75mm	Emergency procedures, unable to meet study requirements, participation in other clinical trial, pregnancy, not a standard CABG or concomitant valve surgery, history of any cardiac surgery other than PTCA and stent placement, history of IABP within the last 30 days, congestive heart failure or NYHA IV in the last 30 days, history of bleeding disorder or thromboembolic disease requiring anticoagulation therapy, hemodynamical instability, history or acute or chronic dialysis, creat > 200 mmol/ml in the last 30 days, documented or suspected acute systemic infection, need for immunosuppressive therapy, CVA within the last 2 weeks, allergy or other contraindication for aspirin or other anticoagulants
Balkhy 2018	C-port	NRCT (historical control)	Not blinded	Not randomized	Patients receiving at least 1 C-port in routine CABG, informed consent, able to make follow-up appointments and examinations, age < 80, LVEF > 30%, life expectancy > 1year.	Participation in other conflicting clinical trials, unable to meet study requirements, pregnancy, preoperative IABP, bleeding disorder or thromboembolic disease requiring anticoagulants, NYHA IV, contraindication to Aspirin, previous CABG.
Cheng 2021	U-clip	NRCT	Not blinded	Not randomized	Single LAD-disease, single RCA-disease, multiple vessel CAD with involvement of LAD and possibility to PCI the non-LAD lesions.	History of left lung trauma or surgery, LVEF < 30%, requirement of emergency CABG, presence of severe COPD with inability of single-lung ventilation.

Abbreviations: St. Jude DAD, St. Jude distal anastomotic device ; NRCT, non-randomized controlled trial ; CABG, coronary artery bypass grafting ; OD, outer diameter ; ID, inner diameter ; RCT, randomized controlled trial ; LVEF, left ventricular ejection fraction ; MVP, magnetic vascular positioner ; CAD, coronary artery disease ; MRI, magnetic resonance imaging ; AADD, automated anastomotic distal device ; CPB, cardiopulmonary bypass ; CAC, coronary anastomotic coupler ; CAG, coronary angiography ; VT, ventricular tachycardia ; VF, ventricular fibrillation ; HIV, human immunodeficiency virus ; IABP, intra-aortic balloon pump ; PTCA, percutaneous transluminal coronary angioplasty ; CVA, cerebrovascular event ; LAD, left anterior descending coronary artery ; RCA, right coronary artery ; PCI, percutaneous coronary intervention ; COPD, chronic obstructive pulmonary disease. -, not described.

Table S4. Patient demographics.

Study	Device	*N* Patients	Age	Male gender	BMI	Hypertension	History of Smoking	Diabetes Mellitus	Peripheral Artery Disease	Previous CVA or TIA	Previous MI	COPD	AF
		S/HS	S/HS	S/HS	S/HS	S/HS	S/HS	S/HS	S/HS	S/HS	S/HS	S/HS	S/HS
Eckstein 2002	St Jude DAD	14	63 (8)	-	-	-		-		-	-		
Wiklund 2005	St Jude DAD	30/30	69 (48-83) / 67 (42-81)[a]	29 (95%) / 26 (85%)	-	19 (63.3%) / 13 (43.3%)	12 (40%) / 12 (40%)	6 (20%) / 9 (30%)	0 (0%) / 4 (13.3%)	4 (13.3%) / 2 (6.7%)	14 (46.7%) / 12 (40%)	0 (0%) / 3 (10%)	-
Klima 2003	MVP	32	65 (9)	27 (90%)	-	23 (71.9%)	15	10 (31.3%)	8	5 (15.6%)	-	5	-
Vicol 2006	MVP	11	-	10 (91%)	-	-	-	-	-	-	-	-	-
Kim 2004	AADD	14	65 (7)	10 (71%)	-	-	8 (57.1%)	8 (57.1%)	-	2 (14.3%)	-	-	-
Klima 2005	CAC	15	65.9 (8.6)	13 (87%)	-	10 (69.2%)	4 (26.7)	-	-	-	6 (38.4%)	-	-
Boening 2005	CAC	46	63.3 (7.5)	44 (96%)	-	-	14 (36.8%)	-	4 (10.2%)	1 (2.6%)	16 (42.1%)	-	3 (7.9%)
Cai 2007	C-port	50	68.0 (9.7)	-	-	-	-	-	-	-	-	-	-
Verberkmoes 2013	C-port	35/36	67.6 (5.6) / 66.5 (5.7)	31 (89%) / 32 (89%)	27.5 (3.7) / 27.1 (3.3)	20 / 20	-	9 / 11	6 / 5	4 / 4	9 / 15	3 / 5	-
Balkhy 2018	C-port	117/3014	-	-	-	49 / 2263	35 / 690	48 / 1139	12 / 369	11 / 382	50 / 609	-	-
Cheng 2021	U-clip	126/154	59.1 (9.0) / 60.7 (10.2)	105 (83%) / 120 (77.9%)	(24.6 (3.5) / 25.3 (3.1)	73 (57.9%) / 92 (59.7%)	62 (49.2%) / 77 (50%)	37 (29.4%) / 43 (27.9%)	-	15 (11.9%) / 21 (13.6%)	21 (16.7%) / 27 (17.5%)	-	-

Table S4. Patient demographics, continued.

Study	Device	*N* Patients	LVEF	NYHA 1-2	NYHA 3-4	EURO-score II	SYNTAX-score	*N* diseased coronary arteries	Previous PCI
		S/HS	S/HS	S/HS	S/HS	S/HS	S/HS	S/HS	S/HS
Eckstein 2002	St Jude DAD	14	-	-	-	-	-	-	-
Wiklund 2005	St Jude DAD	30/30	-	27 / 27	2 / 2	-	-	-	0 (0%) / 1 (3%)
Klima 2003	MVP	32	-	21	10	-	-	-	-
Vicol 2006	MVP	11	-	-	-	-	-	-	-
Kim 2004	AADD	14	58 (7)	-	-	-	-	-	-
Klima 2005	CAC	15	60.6 (10.3)	6	9	-	-	-	4 (26.7%)
Boening 2005	CAC	46	60.6 (12.4)	-	-	-	-	-	11 (28.9%)
Cai 2007	C-port	50	53.8 (10.6) / 50.5 (13.0)	-	-	-	-	-	-
Verberkmoes 2013	C-port	35/36	*32 (91%) / 33 (92%)	-	-	-	-	2.63 (0.49) / 2.61 (0.54)	-
Balkhy 2018	C-port	117/3014	54 / -	-	-	-	-	-	-
Cheng 2021	U-clip	126/154	63.0 (7.1) / 62.5 (6.9)	108 (85.7%) / 126 (81.8%)	18 (14.3%) / 28 (18.2%)	1.4 (0.6) / 1.5 (0.7)	-	-	14 (11.1%) / 17 (11%)

Data is presented for the sutureless (S) and hand-sewn (HS) patients. Single values indicate in-patient controls and belong to the sutureless group. Abbreviations: CVA, cerebrovascular event ; TIA, transient ischemic attack ; MI, myocardial infarction ; COPD, chronic obstructive pulmonary disease ; AF, atrial fibrillation ; LVEF, left ventricular ejection fraction ; PCI, Percutaneous coronary intervention. -, not described. * Number of patients with LVEF > 50%.

Table S5. Interventional details.

Study	Device	*N* Patients	Off-pump	Surgical approach	Coronary target	Donor graft	Graft routing	Coronary target diameter, in mm	Donor graft diameter, in mm
		S/HS	S/HS	S/HS	S/HS	S/HS	S/HS	S/HS	S/HS
Eckstein 2002	St Jude DAD	14	0 (0%)	-	RCA,PDA,OM / -	SVG	-	-	-
Wiklund 2005	St Jude DAD	30 / 30	0 (0%) / 0 (0%)	-	OM,RCA,PDA,D,MR / OM,RCA,PDA,D,MR	SVG / SVG	Single / Single	-	-
Klima 2003	MVP	32	6 (18.8%)	-	RCA,PDA,PLA,LAD,D,OM,MR / -	LIMA, SVG / -	-	-	-
Kim 2004	MVP	11	32 (100%)	Sternotomy	D,OM,PDA,RCA,PLA / LAD,D,OM	IMA,RGEA,SVG / IMA,RGEA,SVG	Single, sequential / Single, sequential	1.0 (0.3) / -	3.0 (0.5) / -
Boening 2005	AADD	14	0 (0%)	Sternotomy	non-LAD / -	SVG / -	Single / -	-	-
Klima 2005	CAC	15	0 (0%)	-	D,RCA,Cx,OM / -	SVG	-	5 (31%)*	-
Vicol 2006	CAC	46	-	Sternotomy	LAD,D,RCA,OM / -	IMA,SVG,RA / -	-	2.03	-
Cai 2007	C-port	50	46 (92%) / -	-	D,OM,PDA / D,OM,PDA	SVG / SVG	-	-	-
Verberkmoes 2013	C-port	35/36	5 (14%) / 3 (8%)	Sternotomy / Sternotomy	non-LAD / non-LAD	SVG / SVG	Single, sequential / Single, sequential	1.48 (0.22) / 1.49 (0.32)	-
Balkhy 2018	C-port	117/3014	116 (99%) / -	TECAB / -	LAD,Cx,RCA / -	SVG / SVG	-	1.8 (0.4) / -	-
Cheng 2021	U-clip	126/154	126 (100%) / 154 (100%)	TECAB / thoracotomy	LAD / LAD,D,RCA	LIMA / LIMA,RIMA	Single / Single, sequential	-	-

Table S5. Interventional details, continued.

Study	Device	*N* Patients	Cross-clamp time, in minutes	CPB-time, in minutes	Anastomotic construction time, in minutes	Blood transfusion rate, in Units
		S/HS	S/HS	S/HS	S/HS	S/HS
Eckstein 2002	St Jude DAD	14	-	-	6-10	-
Wiklund 2005	St Jude DAD	30/30	37 (2) / 35 (2)	65 (4) / 59 (3)	2.3 (0.18)*	-
Klima 2003	MVP	32	46 (19)	92 (16)	2.2 (1.3)	-
Kim 2004	MVP	11	NA	NA	2.9 (0.7) / -	-
Boening 2005	AADD	14	40 (13)	80 (21)	6.6 (5.5) / -	-
Klima 2005	CAC	15	39.4 (9.2)	-	118.3 (48.9) / -	-
Vicol 2006	CAC	46	-	-	-	-
Cai 2007	C-port	50	NA / -	NA / -	-	-
Verberkmoes 2013	C-port	35/36	46 (16) / 44 (18)	81 (28) / 71 (24)	-	-
Balkhy 2018	C-port	117/3014	NA	NA	-	-
Cheng 2021	U-clip	126/154	NA	NA	9.6 (3.7) / 16.9 (3.8)	0.8 (1.6) / 0.9 (1.7)

Data is presented for the sutureless (S) and hand-sewn (HS) patients. Single values indicate in-patient controls and belong to the sutureless group. Abbreviations: St Jude DAD, St Jude distal anastomotic device ; RCA, right coronary artery ; PDA, posterior descending coronary artery ; OM, obtuse marginal coronary artery ; D, diagonal coronary artery ; MR, ramus marginalis coronary artery ; SVG, saphenous vein graft ; MVP, magnetic vascular positioner ; PLA, posterolateral coronary artery ; LAD, left anterior descending coronary artery ; LIMA, left internal mammary artery ; IMA, internal mammary artery ; RGEA, gastro-epoploic artery graft ; AADD, automated anastomotic distal device ; CAC, coronary anastomotic coupler ; RA, radial artery ; Cx, circumflex coronary artery ; RIMA, right internal mammary artery ; CPB, cardiopulmonary bypass. -, not described. NA, not applicable. *Presented without connector deployment time.

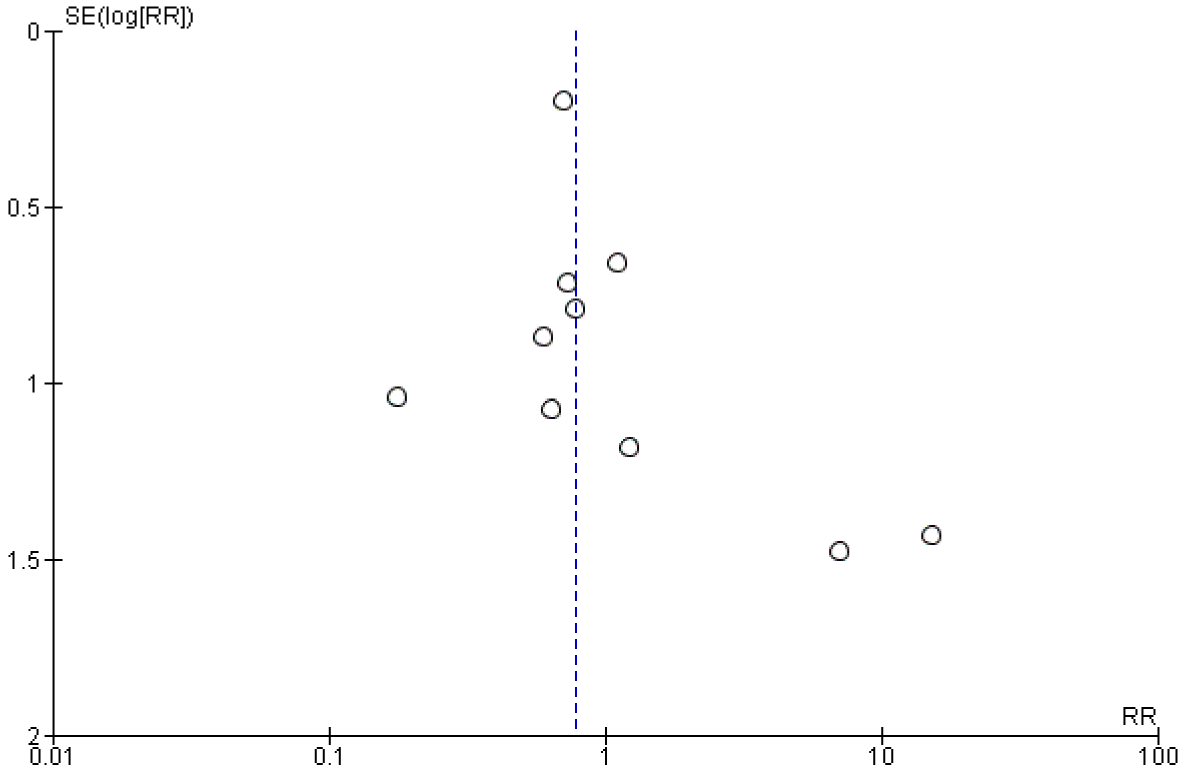

Figure S1. Funnel plot presenting no publication bias for the primary outcome patency.

	sutureless		hand-sewn			Risk Ratio	
**Study or Subgroup**	**Events**	**Total**	**Events**	**Total**	**Weight**	**IV, Random, 95% CI**	**Year**
**3.2.1 <3 months follow-up**							
Eckstein 2002	0	14	0	40		Not estimable	2002
Klima 2003	2	31	6	72	4.8%	0.77 [0.17, 3.63]	2003
Kim 2004	1	14	2	34	2.1%	1.21 [0.12, 12.34]	2004
Boening 2005	1	30	7	37	2.7%	0.18 [0.02, 1.35]	2005
**Subtotal (95% CI)**		**89**		**183**	**9.7%**	**0.56 [0.19, 1.66]**	
Total events	4		15				
Heterogeneity: Tau² = 0.00; Chi² = 1.83, df = 2 (P = 0.40); I² = 0%							
Test for overall effect: Z = 1.04 (P = 0.30)							
**3.2.2 => 3 months follow-up**							
Klima 2005	1	15	4	38	2.6%	0.63 [0.08, 5.21]	2005
Wiklund 2005	7	23	0	23	1.5%	15.00 [0.91, 248.21]	2005
Vicol 2006	3	18	0	18	1.4%	7.00 [0.39, 126.48]	2006
Cai 2007	3	45	2	18	3.9%	0.60 [0.11, 3.30]	2007
Verberkmoes 2013	4	29	4	32	6.8%	1.10 [0.30, 4.01]	2013
Balkhy 2018	22	134	657	3026	68.5%	0.76 [0.51, 1.11]	2018
Cheng 2021	3	107	5	131	5.7%	0.73 [0.18, 3.00]	2021
**Subtotal (95% CI)**		**371**		**3286**	**90.3%**	**0.89 [0.55, 1.44]**	
Total events	43		672				
Heterogeneity: Tau² = 0.06; Chi² = 6.81, df = 6 (P = 0.34); I² = 12%							
Test for overall effect: Z = 0.47 (P = 0.64)							
**Total (95% CI)**		**460**		**3469**	**100.0%**	**0.80 [0.57, 1.12]**	
Total events	47		687				
Heterogeneity: Tau² = 0.00; Chi² = 9.08, df = 9 (P = 0.43); I² = 1%							
Test for overall effect: Z = 1.30 (P = 0.20)							
Test for subgroup differences: Chi² = 0.58, df = 1 (P = 0.45), I² = 0%							

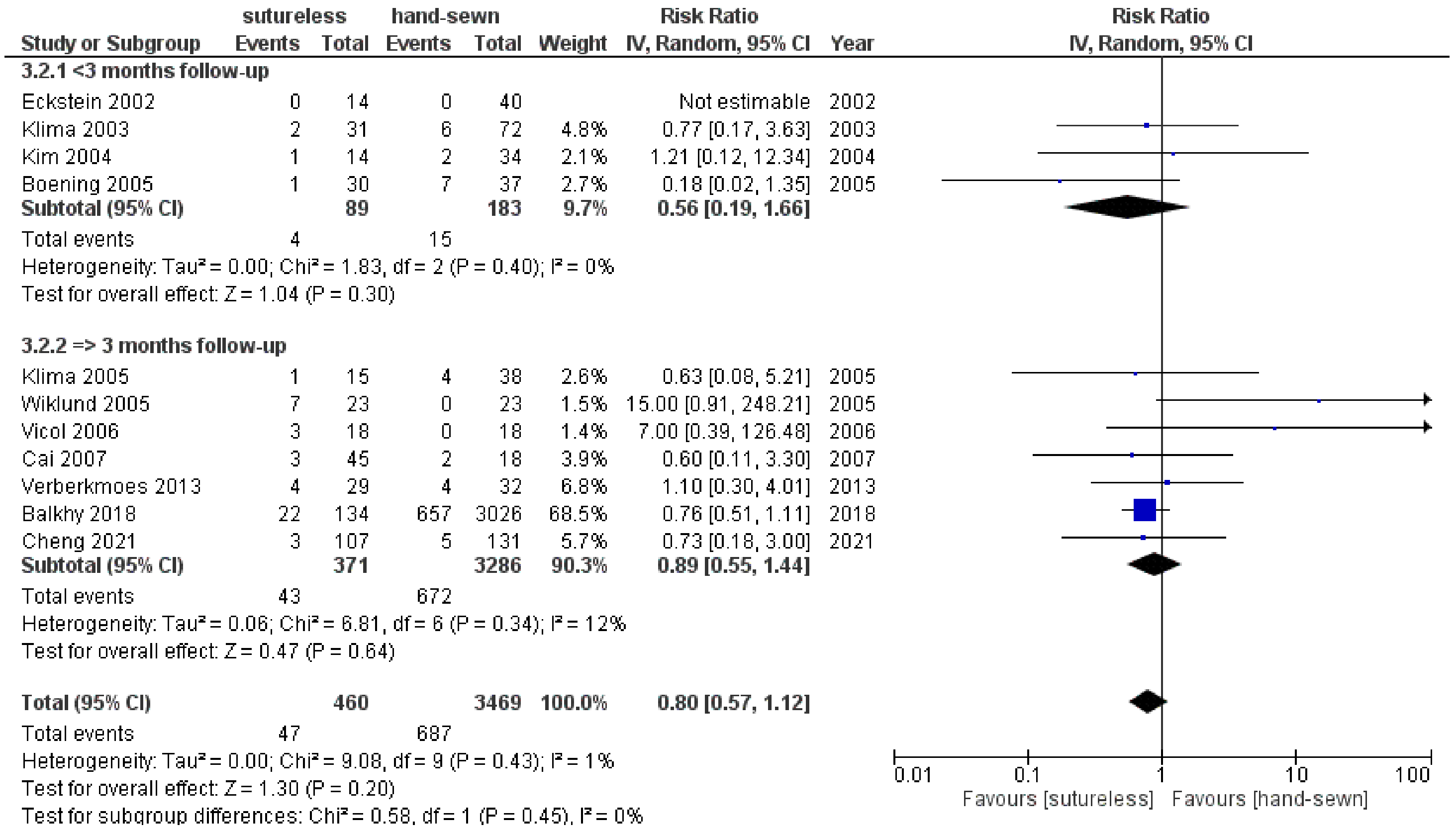

Figure S2. Stratification patency outcome, per follow-up length.

Study or Subgroup	sutureless Events	sutureless Total	hand-sewn Events	hand-sewn Total	Weight	Risk Ratio IV, Random, 95% CI	Year
**3.1.1 Randomized Controlled Trial**							
Wiklund 2005	7	23	0	23	1.4%	15.00 [0.91, 248.21]	2005
Verberkmoes 2013	4	29	4	32	6.5%	1.10 [0.30, 4.01]	2013
**Subtotal (95% CI)**		**52**		**55**	**7.9%**	**2.99 [0.25, 35.80]**	
Total events	11		4				
Heterogeneity: Tau² = 2.16; Chi² = 2.74, df = 1 (P = 0.10); I² = 64%							
Test for overall effect: Z = 0.86 (P = 0.39)							
**3.1.2 Non-Randomized Controlled Trial**							
Eckstein 2002	0	14	0	40		Not estimable	2002
Klima 2003	2	32	6	72	4.6%	0.75 [0.16, 3.52]	2003
Kim 2004	2	31	6	72	4.6%	0.77 [0.17, 3.63]	2004
Klima 2005	1	15	4	38	2.5%	0.63 [0.08, 5.21]	2005
Boening 2005	1	30	7	37	2.6%	0.18 [0.02, 1.35]	2005
Vicol 2006	3	18	0	18	1.3%	7.00 [0.39, 126.48]	2006
Cai 2007	4	45	2	18	4.2%	0.80 [0.16, 3.99]	2007
Balkhy 2018	22	143	657	3026	67.0%	0.71 [0.48, 1.05]	2018
Cheng 2021	3	107	5	131	5.5%	0.73 [0.18, 3.00]	2021
**Subtotal (95% CI)**		**435**		**3452**	**92.1%**	**0.71 [0.51, 1.00]**	
Total events	38		687				
Heterogeneity: Tau² = 0.00; Chi² = 4.25, df = 7 (P = 0.75); I² = 0%							
Test for overall effect: Z = 1.99 (P = 0.05)							
**3.1.3 In-patient Control**							
Eckstein 2002	0	14	0	40		Not estimable	2002
Klima 2003	2	32	6	72	0.0%	0.75 [0.16, 3.52]	2003
Kim 2004	2	31	6	72	0.0%	0.77 [0.17, 3.63]	2004
Boening 2005	1	30	7	37	0.0%	0.18 [0.02, 1.35]	2005
Klima 2005	1	15	4	38	0.0%	0.63 [0.08, 5.21]	2005
Vicol 2006	3	18	0	18	0.0%	7.00 [0.39, 126.48]	2006
Cai 2007	4	45	2	18	0.0%	0.80 [0.16, 3.99]	2007
**Subtotal (95% CI)**		**0**		**0**		**Not estimable**	
Total events	0		0				
Heterogeneity: Not applicable							
Test for overall effect: Not applicable							
**3.1.4 Historical Control**							
Balkhy 2018	22	143	657	3026	0.0%	0.71 [0.48, 1.05]	2018
**Subtotal (95% CI)**		**0**		**0**		**Not estimable**	
Total events	0		0				
Heterogeneity: Not applicable							
Test for overall effect: Not applicable							
**3.1.5 Non-Randomized Controlled Trial**							
Cheng 2021	3	107	5	131	0.0%	0.73 [0.18, 3.00]	2021
**Subtotal (95% CI)**		**0**		**0**		**Not estimable**	
Total events	0		0				
Heterogeneity: Not applicable							
Test for overall effect: Not applicable							
**Total (95% CI)**		**487**		**3507**	**100.0%**	**0.76 [0.55, 1.06]**	
Total events	49		691				
Heterogeneity: Tau² = 0.00; Chi² = 9.05, df = 9 (P = 0.43); I² = 1%							
Test for overall effect: Z = 1.60 (P = 0.11)							
Test for subgroup differences: Chi² = 1.26, df = 1 (P = 0.26), I² = 20.4%							

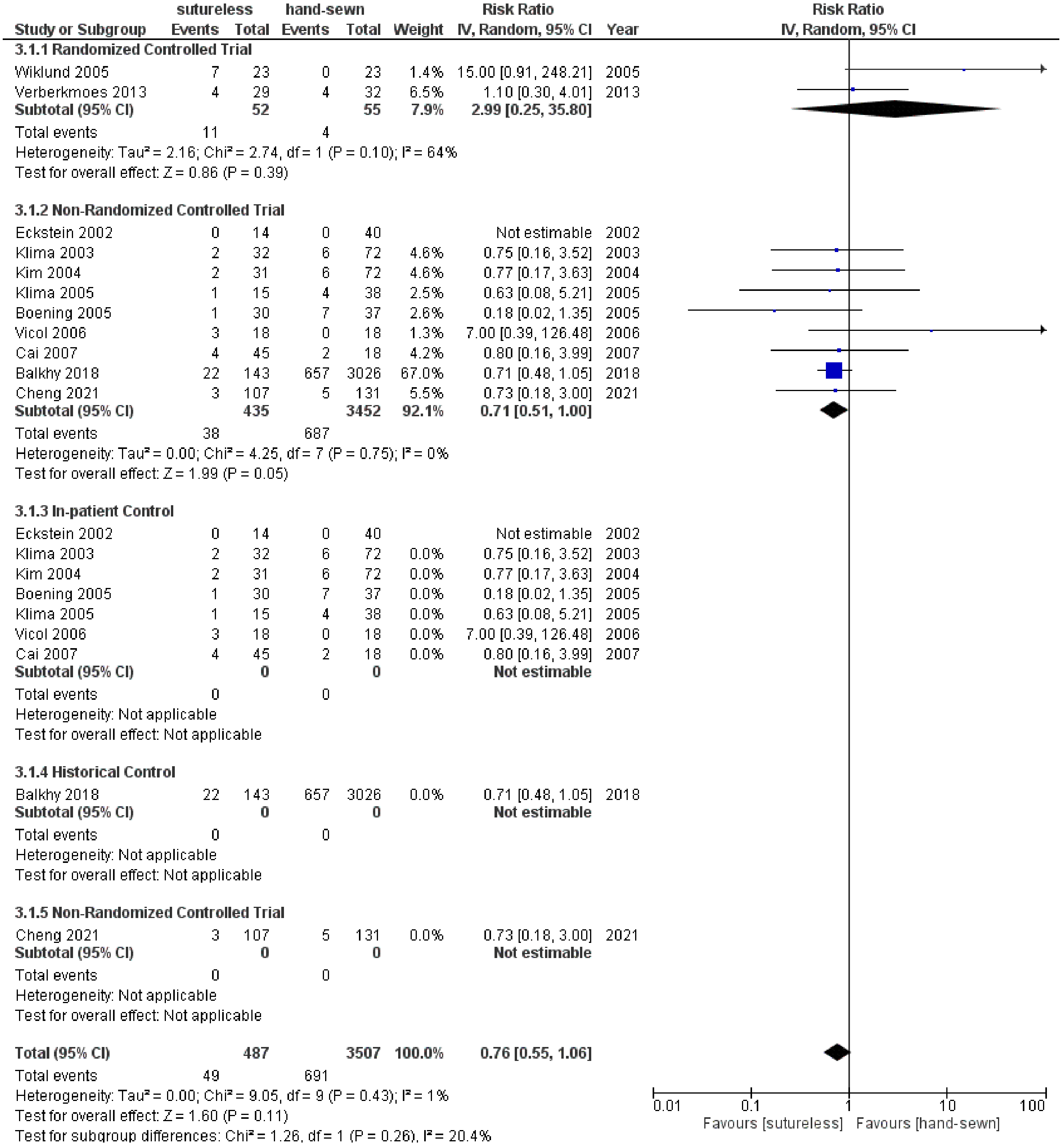

Figure S3. Sensitivity analyses patency outcome, per study design.
